# Molecular epidemiology and phylogenetic analysis of feline calicivirus in Kunshan, China

**DOI:** 10.1186/s12985-024-02319-9

**Published:** 2024-02-27

**Authors:** Semin Kim, Yixi Cheng, Zhenkun Fang, Xinyue Liu, Qiu Zhongqi, Yu Weidong, Aysun Yilmaz, Huseyin Yilmaz, Sajid Umar

**Affiliations:** 1 Global Health Research Center (GHRC), Duke Kunshan University, Suzhou, China; 2 Simba Pet Hospital, Tinglin Park Branch), Maanshan Road, 215335 Kunshan, Suzhou, Jiangsu Province China; 3 Play Pi Kangkang Pet Hospital, Kunshan City Development Zone, 215300 Kunshan, Suzhou, Jiangsu Province China; 4 Department of Virology, Veterinary Faculty, Istanbul University-Cerrahpasa, 35500 Büyükcekmece, Istanbul, Turkey; 5 Division of Natural & Applied Sciences (DNAS), Duke Kunshan University, Suzhou, China

**Keywords:** Cats, China, Epidemiology, Feline calicivirus, Genetic diversity, Phylogenetic Analysis

## Abstract

Feline calicivirus (FCV) is a highly contagious virus in cats, which typically causes respiratory tract and oral infections. Despite vaccination against FCV being a regular practice in China, new FCV cases still occur. Antigenic diversity of FCV hinders the effective control by vaccination. This is first report which aims to investigate the molecular epidemiology and molecular characteristics of FCV in Kunshan, China. The nasopharyngeal swabs were collected from cats showing variable clinical signs from different animal clinics in Kunshan from 2022 to 2023. Preliminary detection and sequencing of the FCV capsid gene were performed to study genetic diversity and evolutionary characteristics. FCV-RNA was identified in 52 (26%) of the samples using RT-PCR. A significant association was found between FCV-positive detection rate, age, gender, vaccination status and living environment, while a non-significant association was found with breed of cats. Nucleotide analysis revealed two genotypes, GI and GII. GII predominated in Kunshan, with diverse strains and amino acid variations potentially affecting vaccination efficacy and FCV detection. Notably, analysis pinpointed certain strains’ association with FCV-virulent systemic disease pathotypes. This investigation sheds light on FCV dynamics, which may aid in developing better prevention strategies and future vaccine designs against circulating FCV genotypes.

## Introduction

Feline calicivirus (FCV), a member of the *Caliciviridae* family and *Vesivirus* genus, is a highly contagious pathogen affecting both domestic cats and wild felines worldwide [1]. FCV was first reported in 1957 and has since been isolated in many countries in Asia, America, and Europe [2–4]. Typical clinical syndromes of FCV infection include upper respiratory tract disease (URTD), oral ulcers, conjunctivitis, rhinitis, fever, diarrhea, and lethargy [5–7]. Without vaccine protection kittens are more susceptible to severe pneumonia following FCV infection [1, 8]. Nevertheless, new virulent mutants of FCV (VS-FCVs) have become increasingly prevalent worldwide in recent years [4, 9]. In contrast to classical FCV infection, VS-FCVs is associated with severe virulent systemic diseases (VSD), including persistent fever, limb edema, bronchitis, and pneumonia [4, 10]. Even adult or vaccinated cats can be infected by these strains and die [1, 4, 11]. Even months or years after recovery from FCV infection, these asymptomatic carrier cats can shed the virus, leading to more epidemics of FCV [12, 13].

The FCV virus has a small, non-enveloped, single-stranded RNA genome approximately 7700 bp in length. Its genome of positive polarity allows the virus to evolve quickly, and its structure, which lacks a lipid envelope, helps it persist in the environment for a relatively long time [1]. FCV contain three functional open reading frames (ORFs): ORF1, ORF2, and ORF3. ORF1 encodes a polyprotein that is post-translationally cleaved into non-structural proteins such as proteases and polymerase [1]. ORF2 encodes a polyprotein that is subsequently processed to produce the major capsid protein (VP1), and can be divided into six regions, A to F, based on their amino acid sequence variability [1, 14]. ORF3 encodes the smaller capsid protein (VP2), which is vital for replication, viral particle assembly, and delivery of the FCV genome into its host cells [15]. In ORF2, genomic regions A, B, D, and F are relatively conserved, whereas regions C and E are more variable [14].

The VP1 protein is the main component of structural proteins in FCV. More importantly, the variable regions of VP1 have one of the highest rates of molecular evolution ever recorded [12]. Conventionally, phylogenetic analysis was performed on VP1 gene [10]. Considering the genetic diversity of the VP1 gene, FCVs worldwide can be classified into two groups: genogroup I and genogroup II [5, 16]. According to previous studies, most FCV strains found in China belong to genogroup II [11]. Therefore, to gain a better understanding of the epidemiology and pathogenesis of FCV it is vital to understand the genetic diversity and characterization of VP1.

In addition, the study of capsid proteins can largely contribute to the development of effective FCV vaccines. The best defense against this virus has always been vaccination. However, with an increasing number of new FCV variant strains frequently emerging in recent years, traditional and existing vaccines are no longer able to provide sufficient protection against them [10]). The aim of this investigation was to explore the epidemiological status and molecular characteristics of FCV in Kunshan, Jiangsu Province, China, as well as to further improve the theoretical basis for the development of new FCV vaccines and better strategies for the prevention and control of FCV. The situation of FCV in Kunshan has not been reported before; thus, this is the first report on the genetic and phylogenetic analysis of FCV strains in Kunshan, China.

## Materials and methods

### Sample collection

**Fig. 1:**
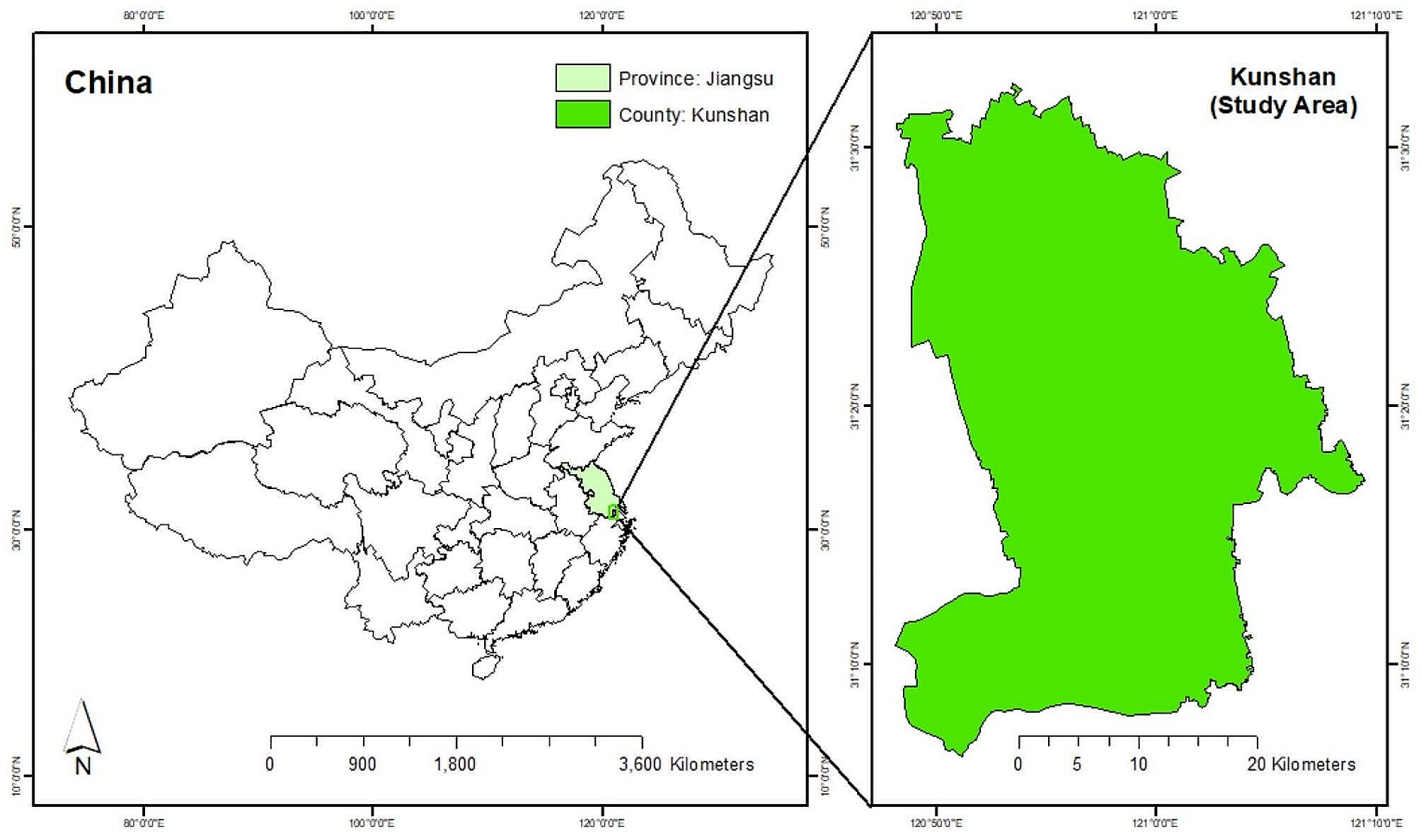
Map showing sampling sites in Kunshan, China

Ethical approval from the Institutional Animal Care and Use Committee (IACUC) was not needed for this study as long as the animals investigated in this study were not identifiable in the retrospective records. A total of 200 nasopharyngeal swab samples were collected from cats using sterile cotton swabs from nine animal clinics in Kunshan from June 2022 to June 2023 (Fig. 1). Nasopharyngeal swab from a single cat were put into one 5 ml sterile tube containing Dulbecco’s Modified Eagle’s Medium (DMEM, Thermo Fisher Scientific, San Jose, CA, USA). Clinic staff recorded data on the breed, age, sex, clinical symptoms, residence, and vaccination status of all the sampled cats. The samples were then immediately transported on wet ice to the Laboratory. All specimens were stored at -80 °C for subsequent RT-PCR and sequence analysis.

### RNA extraction and RT-PCR screening for FCV

RNA was extracted from the swabs, and complementary DNA (cDNA) strands were synthesized from RNA templates using commercially available kits for viral RNA extraction and reverse transcription (TaKaRa MiniBEST Viral RNA/DNA Extraction Kit, Takara, Dalian, China). Preliminary screening of FCV in the samples was performed using FCV-F1 (5′-GTTGACCCTTACTCATACAC-3′) and FCV-R1 (5′-CCCTGGGGTTAGGCGC-3′) as previously described [17]. RT-PCR was performed to amplify and detect a small segment of the VP1 gene using the above-mentioned primers, 2x rapid Taq master mix (Cat# P222, Vazyme, Nanjing, China). and LifeEco Bioer Thermal Cycler (LifeEco TC 96, Bioer, Hangzhou, China). The following thermocycling conditions were used for amplification: 95 °C for 5 min, followed by 40 cycles at 95 °C for 30 s, 52 °C for 30 s, and 72 °C for 30 s, and a 5 min final extension at 72 °C. PCR amplicons were analyzed on 2% agarose gels and approximately 132 bp of PCR amplicons were observed using the GenoSens 1880 gel imaging analysis system (GenoSens 1880, Clinx, Shanghai, China). FCV positive specimens were also tested for coinfection of feline herpesvirus.

### VP1 gene sequencing for mutation detection

To enhance the specificity of the PCR, we slightly modified previously reported primer combinations that amplified the partial length of ORF2 (1980 bp) [12, 17]. The forward and reverse primers were 5′-CCTHCACTGTGATGTGTTCGA-3′ and 5′-GAATTCCCATGTAGGAGGC-3′, respectively. The VP1 gene of the positive samples was amplified using the following protocol: 95 °C for 3 min, 45 cycles of 95 °C for 30 s, 58 °C for 30 s, 72 °C for 2 min, and 72 °C for 10 min. All PCR runs contained a negative template control (nuclease-free water) and a corresponding synthetic positive control sample. PCR was performed using a two-step RT-PCR kit (Takara, Dalian, China). Positive PCR products were sequenced using Sanger sequencing methods.

### Sequence analysis

Nucleotide sequences were analysed, edited, and aligned for phylogenetic analysis using Bio Edit Software version 7.2 9 (Ibis Biosciences, Carlsbad, CA, USA). Sequence electropherograms were carefully analysed, and nucleotide ambiguities were excluded. To make the sequencing data more reliable, we aligned the forward and reverse sequences together to generate a consensus sequence. Multiple sequences were assembled and aligned using ClustalW version 2.0. To understand the molecular epidemiology of the identified FCV in this study, reference sequences were retrieved from the National Centre for Biotechnology Information (NCBI) nucleotide database (http://www.ncbi.nlm.nih.gov) to infer the overall detected virus phylogeny as of July 2023. All sequences were trimmed and aligned according to the VP1 gene. Molecular Evolutionary Genetic Analysis (MEGA version 11.0) was used to construct and analyse the phylogenetic tree [18]. Phylogenetic trees were constructed using the Neighbour Joining method with the Kimura 2-parameter model. These sequences were deposited in GenBank (accession numbers OR472393–OR472444).

### Statistical analysis

Association analyses of FCV infection with gender, living environment, breed, vaccination status and age were performed using the Chi-square test in SPSS V. 19.0. A multi-cat environment was defined as the number of cats of ≥ 2. A value of *p* < 0.05 and *p* > 0.05 were considered statistically significant and non-significant, respectively.

## Results

### Detection of FCV, signalment and clinical findings

Of the 200 nasopharyngeal swab specimens, 52 (52/200, 26%) were positive for FCV, while 148 (148/200, 74%) were negative for FCV. Among these 52, 17 (32.7%) were vaccinated cats and 35 (67.3%) were non-vaccinated cats. Among the 52 positive specimens, 45 (86.5%) were from cats aged below 1 year, and 7 (13.5%) were from cats older than 1 year. A total of 24 (46.1%) and 28 (53.9%) specimens were positive for FCV in male (Tom) and female (Queen) cats, respectively (Tables 1 and 2). Detection rates of 63.5% (33/52) and 36.5% (19/52) were observed in cats living in groups and singly, respectively. Various clinical signs were observed in cats, including respiratory distress, sneezing, coughing, lacrimation, nasal discharge, mouth ulcers, anorexia, and fever. The clinical signs such as nasal discharge, sneezing and mouth ulcers were generally more severe in non-vaccinated cats than in vaccinated cats (personal observation). Chi-square test analysis revealed a significant association (*p* < 0.05) between age, gender, living environment and vaccination status with FCV-positive detection rate, while a non-significant association was observed with breed of cats (*p* > 0.05) (Table 2). Eight FCV positive specimens were coinfected with feline herpesvirus. Mucopurulent ocular discharge was an additional clinical sign in coinfected cats and clinical parameters were more severe in mixed infections than in mono-infections.

**Table 1:** Information on the FCV strains identified in this study

Kunshan FCV strains	Time	Age (months)	Sex	breed	Residential density	Clinical signs	Vaccination status	Genotype	GenBank No.
KN-14	2022	2	M	Hybrid	Multiple	Runny nose, coughing, conjunctivitis	No	I	OR472393
KN-20	2022	3	M	Purebred	Multiple	Oral ulceration, salivation, eye lid edema	No	I	OR472394
KN-27	2022	7	F	Purebred	Multiple	Eyes pus, salivation	Yes	I	OR472395
KN-41	2022	4	M	Purebred	Single	Oral ulceration, salivation, conjunctivitis	No	I	OR472396
KN-57	2022	3.5	F	Purebred	Single	Runny nose, coughing, conjunctivitis	No	I	OR472397
KN-60	2022	15	F	Purebred	Multiple	Eyes pus, salivation	Yes	I	OR472398
KN-76*	2022	8	M	Hybrid	Single	Eyes pus, salivation	Yes	I	OR472399
KN-82	2022	3	F	Purebred	Multiple	Runny nose, coughing, conjunctivitis	No	I	OR472400
KN-90	2022	2.5	M	Hybrid	Multiple	Runny nose, coughing, conjunctivitis	No	I	OR472401
KN-94*	2022	3	F	Hybrid	Single	Runny nose, coughing, conjunctivitis	No	I	OR472402
KN-169	2023	9	F	Purebred	Multiple	Oral ulceration, salivation, conjunctivitis	Yes	I	OR472403
KN-175	2023	25	M	Hybrid	Single	Eyes pus, salivation	Yes	I	OR472404
KN-184	2023	18	F	Hybrid	Single	Runny nose, conjunctivitis	Yes	I	OR472405
KN-185*	2023	2.3	M	Purebred	Multiple	Oral ulceration, salivation, conjunctivitis	No	I	OR472406
KN-189	2023	3	F	Purebred	Multiple	Runny nose, coughing, conjunctivitis	No	I	OR472407
KN-07	2022	6	M	Hybrid	Single	Eye pus, oral ulceration	Yes	II	OR472408
KN-11	2022	5	F	Hybrid	Multiple	Eyes pus, salivation	No	II	OR472409
KN-19*	2022	3	M	Purebred	Single	Oral ulceration, salivation, conjunctivitis	No	II	OR472410
KN-31	2022	3.5	F	Hybrid	Multiple	Runny nose, conjunctivitis	No	II	OR472411
KN-33	2022	6	F	Hybrid	Single	Oral ulceration, salivation, conjunctivitis	Yes	II	OR472412
KN-35	2022	4	M	Purebred	Multiple	Oral ulceration, salivation, conjunctivitis	No	II	OR472413
KN-43	2022	2.5	F	Hybrid	Multiple	Runny nose, conjunctivitis	No	II	OR472414
KN-49	2022	13	M	Purebred	Single	Runny nose, coughing, conjunctivitis	Yes	II	OR472415
KN-51	2022	4	M	Purebred	Single	Runny nose, conjunctivitis	No	II	OR472416
KN-59	2022	2	F	Hybrid	Multiple	Runny nose, coughing, conjunctivitis	No	II	OR472417
KN-68*	2022	3.4	F	Purebred	Multiple	Runny nose, conjunctivitis	No	II	OR472418
KN-69*	2022	2.5	F	Hybrid	Multiple	Coughing, conjunctivitis, salivation	No	II	OR472419
KN-75	2022	3	F	Purebred	Single	Eyes pus, salivation	No	II	OR472420
KN-80	2022	4	F	Purebred	Single	Coughing, sneezing, anorexia	No	II	OR472421
KN-81	2022	4.5	M	Purebred	Multiple	Oral ulceration, salivation, conjunctivitis	No	II	OR472422
KN-87	2022	6	M	Purebred	Multiple	Runny nose, conjunctivitis	Yes	II	OR472423
KN-99	2022	5	M	Hybrid	Multiple	Oral ulceration, salivation, conjunctivitis	Yes	II	OR472424
KN-107	2023	3	F	Purebred	Multiple	Coughing, conjunctivitis, salivation	No	II	OR472425
KN-118	2023	2	M	Purebred	Single	Runny nose, conjunctivitis	No	II	OR472426
KN-119	2023	4	F	Hybrid	Multiple	Oral ulceration, salivation, conjunctivitis	No	II	OR472427
KN-127*	2023	3	M	Hybrid	Multiple	Coughing, conjunctivitis, salivation	No	II	OR472428
KN-137	2023	3.5	M	Hybrid	Multiple	Eyes pus, salivation	No	II	OR472429
KN-145	2023	3	F	Purebred	Multiple	Coughing, conjunctivitis, salivation	No	II	OR472430
KN-149	2023	2.4	F	Hybrid	Single	Oral ulceration, salivation, conjunctivitis	No	II	OR472431
KN-156	2023	3	M	Hybrid	Multiple	Coughing, conjunctivitis, salivation	No	II	OR472432
KN-161	2023	3.5	F	Purebred	Multiple	Oral ulceration, salivation, conjunctivitis	No	II	OR472433
KN-162	2023	18	F	Hybrid	Multiple	Coughing, conjunctivitis, salivation	Yes	II	OR472434
KN-164	2023	6	M	Purebred	Single	Oral ulceration, salivation, conjunctivitis	No	II	OR472435
KN-171	2023	16	M	Hybrid	Multiple	Coughing, conjunctivitis, salivation	Yes	II	OR472436
KN-173	2023	22	F	Hybrid	Multiple	Runny nose, conjunctivitis	Yes	II	OR472437
KN-177	2023	10	F	Purebred	Single	Eyes pus, salivation, sneezing	Yes	II	OR472438
KN-181	2023	3	M	Purebred	Multiple	Oral ulceration, salivation, conjunctivitis	No	II	OR472439
KN-183	2023	9	F	Hybrid	Single	Oral ulceration, salivation, conjunctivitis	Yes	II	OR472440
KN-190*	2023	4	F	Purebred	Multiple	Coughing, conjunctivitis, salivation	No	II	OR472441
KN-191	2023	6	M	Hybrid	Single	Runny nose, conjunctivitis	Yes	II	OR472442
KN-192	2023	6	F	Hybrid	Multiple	Oral ulceration, salivation, conjunctivitis	No	II	OR472443
KN-194	2023	5	M	Purebred	Multiple	Coughing, conjunctivitis, salivation	No	II	OR472444

Note: KN19, KN68, KN69, KN76, KN94, KN127, KN185, KN190 showed co-infection with FHV (indicated with asterisk (*)

**Table 2:** Statistical analysis of FCV positive rate

Parameter	Total number of samples	FCV positive number	FCV positive rate (%)	FCV negative number	FCV negative rate (%)	X^2^	*p*
	*n* = 200	52	26%	148	74%		
**Gender**							
Male	121	24	46.1%	97	80.16%	6.052	0. 014
Female	79	28	53.9%	51	64.55%		
**Breed**							
Hybrid cat	83	25	48.07	58	69.87%	1.252	0.263
Purebred cat	117	27	51.92%	90	76.92%		
**Age**							
≤ 1 year	113	45	86.5%	68	60.17%	25.798	< 0.00001
> 1 year	87	7	13.5%	80	91.95%		
**Residential density**							
Single	111	19	17.11%	92	82.88%	10.23	0.001382
Multiple	89	33	37.07%	56	62.92%		
**Vaccination status**							
Vaccinated	127	17	32.7%	110	86.61%	28.776	< 0.00001
Non vaccinated	73	35	67.3%	38	52.05%		

### Nucleotide homology analysis of FCV VP1 gene

All sequences (*n* = 52) were blasted against each other and previously reported sequences from the NCBI GenBank database. These sequences were compared for nucleotide homology within themselves using pairwise alignment using EMBOSS needle software. Pairwise comparative analysis revealed a homology of 69.2–100%, and 79.5–100% for nucleotide and deduced amino acid sequences, respectively, between the 52 FCV strains identified in this study. Several substitutions were observed in all sequences compared to the reference FCV strains (vaccine strain F9/F4/255). Similarly, sequence identity was 72.3–89.0%, and 81.5–93.7% between 52 FCV strains detected in this study and representative reference strains. The 52 new FCV strains detected in this study revealed 69.4–79.8%, 71.9–79.2%, and 73.5–78.4% nucleotide sequence homology, respectively, and 82.6– 90.3%, 80.7–91.4%, and 82.7–87.6% amino acid sequence identity, respectively, when compared with FCV vaccine strains F9 (M86379), F4 (D31836), and 255 (KM111171). FCV strains identified in this study revealed 72.3–83.2% nucleotide sequence identity and 83.7.4–91.5% amino acid sequence homology when compared with VSD (KM111557) representative strains respectively. Similarly, a nucleotide identity of 74.6–85.7% and amino acid sequence homology 84.1.4–93.2% were observed when compared with the ORD (AY560113) representative strain. The specific FCV strains from worldwide that shared the highest nucleotide identity with the 52 Kunshan FCV strains are shown in Table 3.

**Table 3:** Fifty-two (52) Kunshan FCV strains were matched up with other strains worldwide that had been published before in the GenBank database, according to the highest nucleotide similarity

Kunshan strains	NCBI strains	Origin	Sequence ID	Nucleotide similarity (%)
KN-14	GXNN04-20	China	MZ712020.1	100
KN-20	HZ-19	China	MW658487.1	99.89
KN-27	BJ-113	China	MW088952.1	99.83
KN-41	HZ-19	China	MW658487.1	99.89
KN-57	GXNN04-20	China	MZ712020.1	100
KN-60	BJ-113	China	MW088952.1	99.83
KN-76	GXNN04-20	China	MZ712020.1	100
KN-82	HZ-19-5	China	MW658487.1	99.89
KN-90	FCV-HB10	China	OM650777.1	99.72
KN-94	BJ-113	China	MW088952.1	99.83
KN-169	FCV-HB10	China	OM650777.1	99.89
KN-175	FCV-HB10	China	OM650777.1	100
KN-184	FCV-HB10	China	OM650777.1	100
KN-185	FCV-HB10	China	OM650777.1	99.78
KN-189	FCV-HB10	China	OM650777.1	100
KN-07	FCV-SH191	China	OM650791.1	99.83
KN-11	FCV-SH191	China	OM650791.1	99.83
KN-19	FCV-GD383	China	OM650799.1	99.67
KN-31	FCV-HB384	China	OM650800.1	99.83
KN-33	FCV-YN189	China	OM650789.1	99.72
KN-35	FCV-SH191	China	OM650791.1	99.56
KN-43	FCV-SH191	China	OM650791.1	99.83
KN-49	FCV-SH191	China	OM650791.1	99.83
KN-51	FCV-SH191	China	OM650791.1	99.79
KN-59	FCV-SH191	China	OM650791.1	99.83
KN-68	FCV-SH191	China	OM650791.1	99.83
KN-69	FCV-SH191	China	OM650791.1	99.83
KN-75	FCV-SH191	China	OM650791.1	99.83
KN-80	FCV-SH192	China	OM650792.1	99.83
KN-81	FCV-SH191	China	OM650791.1	99.83
KN-87	FCV-SH191	China	OM650791.1	99.83
KN-99	FCV-GD383	China	OM650799.1	99.67
KN-107	FCV-HB384	China	OM650800.1	99.83
KN-118	FCV-YN189	China	OM650789.1	99.72
KN-119	FCV-SH191	China	OM650791.1	99.56
KN-127	FCV-SH191	China	OM650791.1	99.83
KN-137	FCV-SH191	China	OM650791.1	99.83
KN-145	FCV-SH191	China	OM650791.1	99.81
KN-149	FCV-SH191	China	OM650791.1	99.83
KN-156	FCV-SH191	China	OM650791.1	99.83
KN-161	FCV-SH191	China	OM650791.1	99.83
KN-162	FCV-SH191	China	OM650791.1	99.80
KN-164	FCV-SH192	China	OM650792.1	99.83
KN-171	FCV-SH191	China	OM650791.1	99.83
KN-173	FCV-GD383	China	OM650799.1	99.67
KN-177	FCV-SH191	China	OM650791.1	99.56
KN-181	FCV-SH191	China	OM650791.1	99.83
KN-183	FCV-SH191	China	OM650791.1	99.79
KN-190	FCV-SH191	China	OM650791.1	99.83
KN-191	FCV-SH191	China	OM650791.1	99.81
KN-192	FCV-SH191	China	OM650791.1	99.83
KN-194	FCV-SH192	China	OM650792.1	99.83

### Phylogenetic analysis of VP1 gene

**Fig. 2:**
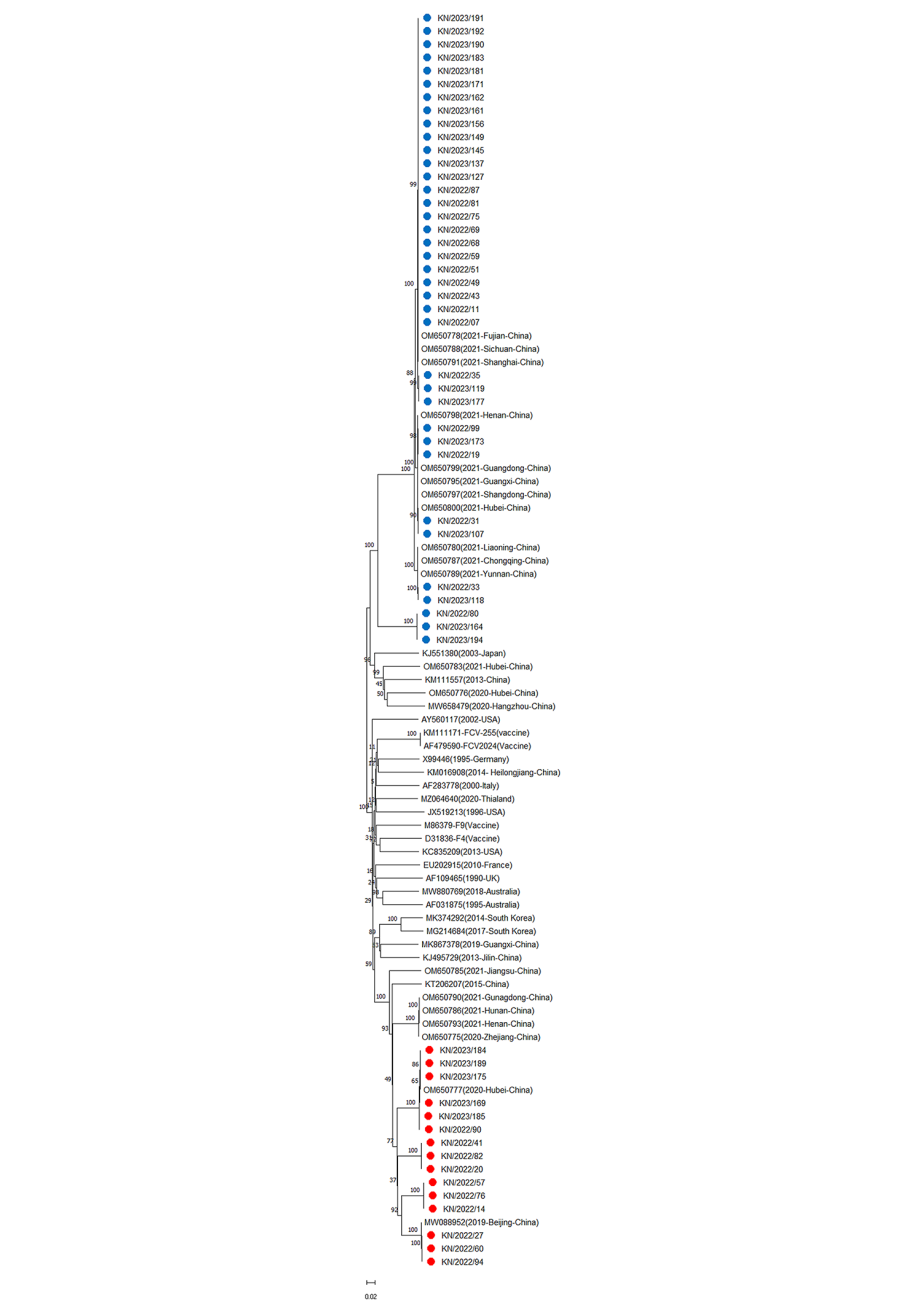
Phylogenetic trees were constructed based on ORF2 in the study. The phylogenetic tree was constructed using the neighbor-joining method for 1000 bootstrap values. The sequences identified in this study were labeled with red (GI) and blue (GII) circles

To reveal the evolutionary characteristics of FCV, we constructed a phylogenetic tree among 52 new FCV strains identified in this study and 43 reference FCV strains downloaded from the GenBank database. Phylogenetic analysis clustered the FCV strains into two genotypes, GI and GII, which are denoted by red and blue circles, respectively. A total of 37 sequences were clustered into the GII genotype clade, whereas only 15 sequences were clustered within the GI genotype clade. FCV-GII strains identified in this study demonstrated the closest genetic relationship with strains reported from different provinces in China (Shanghai, Fujian, Sichuan, Hubei, Shandong, and Nanjing). Interestingly, the FCV-G1 strains identified in this study were more closely related to the FCV strains previously reported in Beijing and Hubei (Fig. 2).

### Deduced amino acid analysis of VP1 gene

Amino acid positions within the hypervariable region E of the VP1 protein were analyzed and compared with those of previously reported reference strains of FCV. ORF2 encodes the VP1 protein, which comprises 668–671 amino acid residues. The amino acid sites between positions 426 and 523 were designated region E of the VP1 protein. FCV GI and GII identified in this study varied at several amino acid sites within region E of the VP1 protein (Table 4). Notably, 52 FCV strains in the present study exhibited multiple site mutations in region E of the VP1 protein when compared with the vaccine strain F9 (M86379)(439 N → S/T, 441T → R/N/S, 446T → I, 448 A → S, 449T → K/N/S, 450G → E/Q, 453T → S/A, 455D → G, 465G → S, 492I → V, 521 K → S/E/T, 522 K → A/D/E/G, and 523 A → V/T). Furthermore, 52 new FCV strains identified in this study were compared with the VSD and ORD reference strains. Different amino acids were detected at seven virulence factor-related loci within the E region of the VP1 protein of 52 isolates (amino acid sites:438, 440, 448, 452,455,465, 492). A comparison of amino acids at seven virulence factor-related sites between FCV genotypes in this study and reference strains (M86379, D31836, KM111171, KM111557, and AY560113) is presented in Table 5.

**Table 4:** Amino acids comparison between GI and GII identified in this study for capsid protein E region of the VP1 protein

Genotype	Amino acids positions of GI and GII identified in this study											
	426	436	460	464	489	490	491	492	500	501	509	512
I	P	A	N	K	T	A	I	V	L	K	T	A
II	G	S	T	R	V	G	T	L/A	I	S	V	V

**Table 5:** Comparison of amino acids at seven virulence factor-related sites between FCV genotypes of this study and reference strains

FCV strains	Genotype	Amino acids at seven virulence factor-related sites						
		438	440	448	452	455	465	492
M86379-F9 (Vaccine)	I	T	G	A	D	D	G	I
D31836-F4 (Vaccine)	I	T	G	A	D	A	G	R
KM111171-255(vaccine)	I	A	G	A	D	T	G	R
VSD-FCV (KM111557)	-	V	Q	K	E	T	S	L
ORD-FCV (AY560113)	-	T	S	P	D	D	G	V
KN-14	I	T	G	A	D	G	S	V
KN-20	I	T	G	A	D	D	S	V
KN-27	I	T	G	A	D	D	S	V
KN-41	I	T	G	A	D	D	S	V
KN-57	I	T	G	A	D	G	S	V
KN-60	I	T	G	A	D	D	S	V
KN-76	I	T	G	A	D	G	S	V
KN-82	I	T	G	A	D	D	S	V
KN-90	I	T	G	S	D	D	S	V
KN-94	I	T	G	A	D	D	S	V
KN-169	I	T	G	S	D	D	S	V
KN-175	I	T	G	S	D	D	S	V
KN-184	I	T	G	S	D	D	S	V
KN-185	I	T	G	S	D	D	S	V
KN-189	I	T	G	S	D	D	S	V
KN-07	II	T	G	A	D	D	S	I
KN-11	II	T	G	A	D	D	S	I
KN-19	II	T	G	A	D	G	S	I
KN-31	II	T	G	A	D	G	S	I
KN-33	II	T	G	A	D	D	S	I
KN-35	II	T	G	A	D	D	S	I
KN-43	II	T	G	A	D	D	S	I
KN-49	II	T	G	A	D	D	S	I
KN-51	II	T	G	A	D	D	S	I
KN-59	II	T	G	A	D	D	S	I
KN-68	II	T	G	A	D	D	S	I
KN-69	II	T	G	A	D	D	S	I
KN-75	II	T	G	A	D	D	S	I
KN-80	II	T	G	A	D	T	G	I
KN-81	II	T	G	A	D	D	S	I
KN-87	II	T	G	A	D	D	S	I
KN-99	II	T	G	A	D	G	S	I
KN-107	II	T	G	A	D	G	S	I
KN-118	II	T	G	A	D	D	S	I
KN-119	II	T	G	A	D	D	S	I
KN-127	II	T	G	A	D	D	S	I
KN-137	II	T	G	A	D	D	S	I
KN-145	II	T	G	A	D	D	S	I
KN-149	II	T	G	A	D	D	S	I
KN-156	II	T	G	A	D	D	S	I
KN-161	II	T	G	A	D	D	S	I
KN-162	II	T	G	A	D	D	S	I
KN-164	II	T	G	A	D	T	G	I
KN-171	II	T	G	A	D	D	S	I
KN-173	II	T	G	A	D	G	S	I
KN-177	II	T	G	A	D	D	S	I
KN-181	II	T	G	A	D	D	S	I
KN-183	II	T	G	A	D	D	S	I
KN-190	II	T	G	A	D	D	S	I
KN-191	II	T	G	A	D	D	S	I
KN-192	II	T	G	A	D	D	S	I
KN-194	II	T	G	A	D	T	G	I

## Discussion

Respiratory viruses frequently cause severe diseases in cats and have a significant impact on morbidity and mortality worldwide. FCV is a common contagious virus that causes feline infectious diseases with poor outcomes, especially in nonvaccinated cats. Over the past few decades, FCV have become a serious burden to the health of cats [19]. FCV tends to mutate more often than other viruses, mainly because of the lack of exonuclease proofreading activity displayed by their RNA polymerases, which may render vaccines less effective against FCV infections [17, 20]. Therefore, it is imperative to understand the epidemiological patterns and genetic characteristics of FCV in order to implement better therapeutic and preventive measures. Although, FCV has been reported from several regions of China [10–12, 17, 20–24], there are relatively a few published reports on evolutionary characteristics of FCV. From an evolutionary perspective, this study aimed to investigate circulating strains of FCV in Kunshan.

In this study, 200 nasopharyngeal swabs were collected from cats in Kunshan and subjected to FCV detection. Overall, the FCV detection rate was 26%, which was higher than that reported in previous studies conducted in China [21, 24, 25]. In the FCV positive cats, 32.7% were vaccinated, similar to previously reported [11, 17, 26]. However, vaccinated cats generally exhibit reduced clinical signs. This suggests that FCV might be undergoing frequent adaptive mutations, resulting in poor protective efficacy of routine vaccines. There was a significant higher positive rate (*p* < 0.05) among cats living in groups and having less than one year of age, which is consistent with previous studies [3, 11, 17, 27, 28]. However, no significant association (*p* > 0.05) was found between the FCV detection rate and breed of the cats. Notably, eight FCV positive specimens were found coinfected with feline herpesvirus. Other pathogens associated with feline respiratory diseases complex such as *Chlamydia felis* and *Mycoplasma felis* were not tested in this study.

The VP1 protein showed the highest variability and tended to undergo mutations more frequently than the other capsid proteins of FCV. VP1 protein consisted of six regions (A–F). Region E of the VP1 protein is considered hypervariable and contains major targets of virus-neutralizing antibodies (B-cell epitopes) ( [11, 29]. Because of these specific features, VP1 is often targeted for novel vaccine development and clinical diagnosis. Genetic and phylogenetic analyses of the VP1 gene revealed abundant genetic diversity among the FCV strains identified in this study and reference strains. 52 FCV strains in this study clustered into two genotypes, namely genotype I (GI) and genotype II (GII), with 15 (15/52, 28.84%) isolates clustered into the GI clade and 37 (37/52, 71.15%) isolates belonging to the GII clade, indicating that genotype II was the predominant genotype currently circulating in Kunshan City, which is consistent with the latest studies from China [17, 22]. However, this finding differs to some previous studies which reports predominance of genotype I in China [10–12, 25]. We did not notice any obvious link between genotypes and clinical signs, and variable clinical signs were noticed by clinicians in FCV-positive cats [17, 20, 22, 24].

Mutation is an important aspect of the evolutionary process and can lead to genetic variation in FCVs in a field in which the evolutionary process depends. However, it remains unclear whether genetic variations in FCVs alter the immune response to commercial vaccine antigens. Nucleotide and deduced amino acid analysis revealed several mutations in the VP1 capsid gene and genetic divergence of FCVs strains in this study with reference strains, which was in line with previous reports [11, 17, 20, 22]. FCV strains in this study shared 70.8–88.0% nucleotide identity with the reference strains. Notably, all the detected FCV strains in this study showed a distant relationship with vaccine strains (M86379, D31836, and KM111171), suggesting that these vaccines may not provide effective cross-protection against the FCV strains circulating in Kunshan, China. In this study, several amino acid substitutions were observed at seven virulence factor-related positions among the new FCV isolates, VSD, and ORD strains. These mutations may influence antigenicity of the virus by inducing stronger binding or neutralizing antibody responses. In addition, these mutations can assist viruses in adapting to evolutionary pressure. Vaccines appear to exert evolutionary pressure on FCV, resulting in the emergence of new FCV variants. These new FCV genotypes are thought to disrupt vaccine-induced defenses among immunized cats, leading to clinical cases in vaccinated cat populations. The protective efficacy of current vaccines (based on Genotype I of FCV) against genotype II strains in China is unknown; therefore, experimental studies are needed for confirmation. In addition, it is imperative to update vaccine strains on a regular basis according to the circulating genotypes of FCV for better preventive measures.

Our study has some limitations. First, we could not collect specimens from other cities in Jiangsu Province; therefore, a clear prevalence and comparison of FCV genotypes in Jiangsu Province cannot be drawn. Second, due to limited funding, we could not culture FCV isolates or test recombination events among the vaccine and field strains of FCV. Third, we could not perform a serum virus neutralization assay to assess cross-reactivity between different field and vaccine strains.

## Conclusion

In summary, the present study describes the molecular epidemiology and evolutionary characteristics of FCV in Kunshan, China. FCV strains from the present study exhibited high genetic diversity and were grouped into two genotypes, with genotype II being the predominant genotype currently circulating in Kunshan city. Continuous laboratory-based surveillance programs in other parts of China are warranted to provide new insights into the evolutionary characteristics of FCV and develop and implement better vaccination strategies in China.
